# Study of In Vitro and In Vivo Carbamazepine Release from Coarse and Nanometric Pharmaceutical Emulsions Obtained via Ultra-High-Pressure Homogenization

**DOI:** 10.3390/ph13040053

**Published:** 2020-03-26

**Authors:** Juan D. Echeverri, Maria J. Alhajj, Nicolle Montero, Cristhian J. Yarce, Alvaro Barrera-Ocampo, Constain H. Salamanca

**Affiliations:** 1 Programa de Maestría en Formulación de Productos Químicos y Derivados, Facultad de Ciencias Naturales, Universidad Icesi, Calle 18 No. 122-135, Cali 76003, Colombia; juandiegoqf@hotmail.com; 2 Laboratorio de Diseño y Formulación de Productos Químicos y Derivados, Facultad de Ciencias Naturales. Universidad Icesi, Calle 18 No. 122-135, Cali 76003, Colombia; mariajoalhajj@hotmail.com (M.J.A.); nicollemontero76@gmail.com (N.M.); cjyarce@icesi.edu.co (C.J.Y.); 3 Departamento de Ciencias Farmacéuticas, Facultad de Ciencias Naturales, Universidad Icesi, Calle 18 No. 122-135, Cali 76003, Colombia; aabarrera@icesi.edu.co

**Keywords:** carbamazepine, coarse emulsion, in vitro/in vivo drug release, nanoemulsion, ultra-high-pressure homogenization

## Abstract

In the past decade, pharmaceutical nanotechnology has proven to be a promising alternative for improving the physicochemical and biopharmaceutical features for conventional pharmaceutical drug formulations. The goal of this study was to develop, characterize, and evaluate the in vitro and in vivo release of the model drug carbamazepine (CBZ) from two emulsified formulations with different droplet sizes (coarse and nanometric). Briefly, oil-in-water emulsions were developed using (i) Sacha inchi oil, ultrapure water, Tween^TM^ 80, and Span^TM^ 80 as surfactants, (ii) methyl-paraben and propyl-paraben as preservatives, and (iii) CBZ as a nonpolar model drug. The coarse and nanometric emulsions were prepared by rotor–stator dispersion and ultra-high-pressure homogenization (UHPH), respectively. The in vitro drug release studies were conducted by dialysis, whereas the in vivo drug release was evaluated in New Zealand breed rabbits. The results showed that nanoemulsions were physically more stable than coarse emulsions, and that CBZ had a very low release for in vitro determination (<2%), and a release of 20% in the in vivo study. However, it was found that nanoemulsions could significantly increase drug absorption time from 12 h to 45 min.

## 1. Introduction

In the biopharmaceutical classification system (BCS), four categories have been defined for pharmacological active ingredients in relation to their respective physicochemical features, such as solubility and permeability [1,2,3,4]. In accordance with the aforementioned BCS, drugs that are part of category II (high permeability and low solubility in aqueous media) [5,6] have been a subject of great interest for the pharmaceutical sector, which continuously seeks to improve these characteristics through the development of new or improved pharmaceutical dosage forms. Thus, pharmaceutical nanotechnology has proven to be an interesting alternative to enhance the physicochemical and biopharmaceutical properties of such compounds, where the most studied and reported systems involve liposomes [7,8,9,10,11,12,13] and polymeric nanoparticles [14,15,16,17,18]. However, to date, the available information about other types of formulations, such as nanoemulsions, is much more limited [19,20,21,22,23,24,25]. This lack of knowledge may be because such heterodispersed systems are widely known to have thermodynamic problems. Therefore, the development of these heterodispersed formulations is quite complex and usually requires the use of more ingredients and the employment of more unit operations during the manufacturing process. However, these heterodispersed systems have also shown great pharmaceutical benefits, such as enhanced organoleptic characteristics [26,27] and improved physicochemical stability [28,29], and the ability to modify several biopharmaceutical features for a wide range of nonpolar drugs [30,31]. 

Currently, one of the most promising strategies used for the development of emulsified systems on a nanometric scale is ultra-high-pressure homogenization (UHPH) [32]. This technique is based on a high-energy dispersion process, where a conventional emulsion (coarse emulsion) is passed through a micrometric diameter nozzle, causing high turbulence, shear, and cavitation [33]. Consequently, UHPH allows emulsified systems to reach droplet sizes between 10 and 500 nm with very low polydispersity, which has a favorable impact on the physical stability of these formulations [34,35,36,37].

Conversely, it is important to note that the information available about the in vitro and in vivo release of CBZ from emulsified oral formulations with different droplet sizes is very limited. Therefore, the main goals of this study were focused on two aspects: (i) the development and characterization of different emulsified CBZ formulations with different droplet sizes (coarse and nanometric), and (ii) evaluation of the in vitro and in vivo drug release from both emulsified formulations.

## 2. Materials and Methods

### 2.1. Materials

CBZ and sacha inchi oil (SIO) were provided by Tecnoquimicas S.A Pharmaceutical Company (Cali, Valle del Cauca, Colombia) and Nutresacha SA (Santander de Quilichao, Cauca, Colombia), respectively. Sorbitan oleate (Span^TM^ 80, hydrophilic-lipophilic balance (HLB) = 4.3) and Polysorbate 80 (Tween^TM^ 80, HLB = 15) were purchased from Croda (Snaith, UK). Methyl-paraben and propyl-paraben were acquired from Sigma-Aldrich (St. Louis, MO, USA). Water Type II (ultrapure water) was obtained via a Millipore Elix Essential purification system (Merck KGaA, Darmstadt, Germany), while the water employed for high-performance liquid chromatography (HPLC) was purified using a Sartorius Arium-Pro (Sartorius AG, Gotinga, Germany). All solvents were of analytical reagent grade.

### 2.2. SIO/Water Interface Physicochemical Characterization

#### 2.2.1. SIO/Water Partition Assay

The partition study of CBZ between SIO and ultrapure water was conducted in an incubated orbital shaker (Inkubator 1000 with Unimax 1010, Heidolph Instruments, Schwalbach, Germany). For this, 2.4 g CBZ was added to 50 mL of the oil phase (Mixture 1) with a stirring speed of 400 rpm for 45 min at 37 °C. Subsequently, 50 mL of ultrapure water was added to Mixture 1, forming a ternary mixture (CBZ, SIO and ultrapure water) which was stirred at 400 rpm for 45 min at 37 °C. Once the CBZ was distributed in the respective phases, it was left to rest for 3 min, during which a complete separation of SIO and ultrapure water occurred. Immediately after separation, 1 mL aliquots were taken from the oil and water phases (initial time), and the concentrations were determined using a UV/Vis spectrophotometer (UV-1800, Shimadzu, Milton Keynes, UK). Calibration curves of CBZ in SIO and ultrapure water were determined using serial concentrations of 1.0, 3.0, 5.0, 10.0, 15.0, 20.0, and 23.75 mg/mL. Finally, the ternary mixture was left to rest for 8 h (final time), after which 1 mL aliquots were taken from each respective phase and the concentration was measured by UV/Vis spectrophotometry.

#### 2.2.2. SIO/Water Interface Effect

The value of the interfacial tension generated between SIO and ultrapure water (γ_o/w_) was determined using the pendant drop method with a tensiometer (OCA15EC, Dataphysics Instruments, Filderstadt, Germany). Briefly, a drop of the oil was deposited into 3 mL of ultrapure water, where the oil was automatically dispensed from a 500 µL syringe (DS 500GT, Dataphysics Instruments, Filderstadt, Germany) equipped with a needle dispenser (SNC 021/011, Dataphysics Instruments, Filderstadt, Germany), while the water was contained in a flat-faced quartz cell. The instrument recorded video captures with an IDS USB 3 camera (IDS Imaging Development Systems GmbH, Obersulm, Germany) using appropriate software (SCA 21, Dataphysics Instruments, Filderstadt, Germany). Finally, the same procedure was repeated with the incorporation of CBZ into SIO at concentrations of 1.0, 4.0, 8.0, 12.0, and 23.8 mg/mL. Each measurement was conducted in triplicate using freshly prepared samples.

### 2.3. Elaboration of Emulsified Formulations

**Figure 1:**
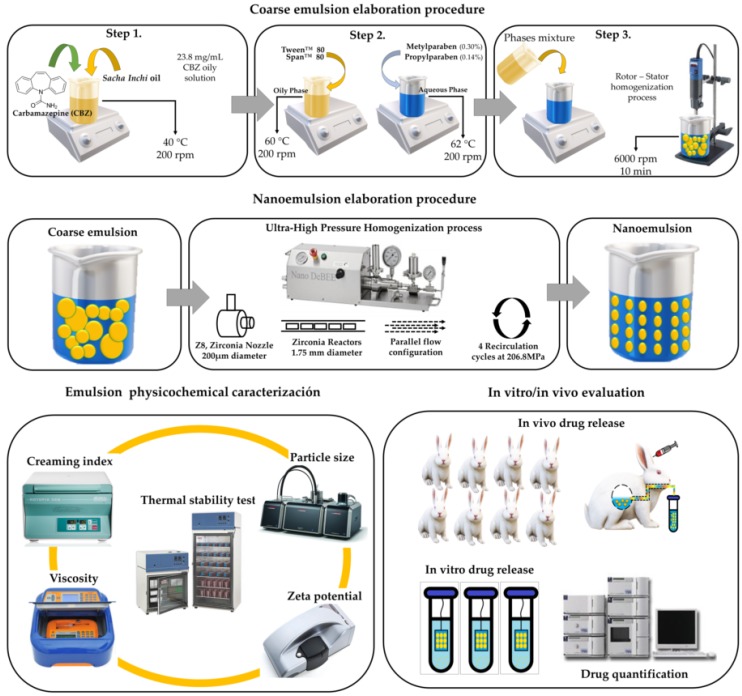
Schematic of the elaboration process, physicochemical characterization, and drug release assays for emulsified carbamazepine (CBZ) formulations.

The schematics of the elaboration process, physicochemical characterization of the emulsified systems, and drug release assays are depicted in Figure 1.

#### 2.3.1. Coarse Emulsions

CBZ was added into SIO at 40 °C under constant stirring (200 rpm) until an oily solution of 23.8 mg/mL was attained. The oily solution and ultrapure water were then heated to 60 °C and 62 °C, respectively, at which point where methyl-paraben and propyl-paraben preservatives were added into the ultrapure water to concentrations of 0.3% *w*/*w* and 0.14% *w*/*w*. In contrast, Tween^TM^ 80 and Span^TM^ 80 surfactants were added into the oily solution at 2% *w*/*w*, providing an HLB value of the surfactant blend of 8, which is, in fact, the required HLB of the oil that leads to high stabilization of such emulsified systems [37,38]. Afterward, the oily phase was poured into the aqueous phase, using an Ultraturrax IKA^®^ at 6000 rpm for 10 min, and the emulsified systems were cooled to room temperature (25 °C) to obtain the coarse emulsions. 

#### 2.3.2. Nanoemulsions 

Once the coarse emulsions were prepared, 600 g measures were subjected to UHPH using a Nano DeBEE Laboratory Homogenizer (BEE international, South Easton, MA, USA), where the operating conditions employed were: zirconia nozzle (Z8) with an orifice diameter of 200 µm, six zirconia reactors with orifice diameter of 1.75 mm, a pressure of 3 × 10^4^ psi (206.8 MPa), and a parallel flow configuration, with a total of four recirculation cycles. These conditions were previously defined by means of a series of tests conducted before the development of the nanoemulsions.

### 2.4. Thermal Stability Assay of Emulsions and Nanoemulsions

The coarse emulsions and nanoemulsions were placed in Falcon™ 15 mL conical centrifuge tubes, which were subsequently left at two temperature conditions (60 °C ± 2 °C and 4.0 °C ± 0.5 °C). For this, each thermal condition was varied every 48 h for 2 weeks, starting at 60 °C for the first 48 h, then at 4 °C, etc. The stability parameters evaluated were creaming index, droplet size, viscosity, zeta potential, electrical conductivity, and pH.

#### 2.4.1. Creaming Index 

In this case, the freshly made coarse emulsion and nanoemulsion were added to Falcon™ 15 mL conical centrifuge tubes (diameter =1.5 cm) and centrifuged at 3000 rpm (150 RFC) for 4 h in a centrifuge (Rotofix 32A, Hetttich GmBH, Tuttlingen, Germany). The value of the creaming index (CI) was determined as

(1) $$CI=\frac{H_{S}}{H_{E}}\times 100$$

where H_S_ is the sediment height and H_E_ is the sample height before centrifugation.

#### 2.4.2. Droplet Size 

For the coarse emulsion, the droplet size distribution was assessed using a laser particle sizer Analysette 22 MicroTec Plus (Fritsch, Idar-Oberstein, Germany) with an automatic analysis setup. First, ~0.5 g of the emulsion was diluted with 10 mL of ultrapure water at 25 °C ± 2 °C and stirred at 400 rpm. The appropriate amount sample was determined as when the obscurance level (between 2% and 8%) was reached in the equipment [39]. For the nanoemulsion, the particle size and the polydispersity index (PDI) were determined using a Zetasizer nano ZSP (Malvern Instrument, Worcestershire, UK), where 10 µL of each sample was taken and dissolved in ~10 mL of ultrapure water. The particle size was measured via dynamic light scattering with an angle scattering of 173° at 25 °C using a quartz flow cell (ZEN0023). In this case, the instrument reported the particle size as having the mean particle diameter (z-average) and PDI ranging from 0 (monodisperse) to 1 (very broad distribution). All measurements were performed in triplicate.

#### 2.4.3. Viscosity

The viscosity was measured using a viscometer (micro-visc, RheoSense Inc., San Ramon, CA, USA), applying a shear rate of 480 s^−1^ and 7850 s^−1^ for the coarse emulsion and nanoemulsion, respectively. All measurements were performed in triplicate.

#### 2.4.4. Zeta Potential, Electrical Conductivity, and pH Measurements

Zeta potential measurements were made using a Zetasizer nano ZSP (Malvern Instruments, Worcestershire, UK) at 25 °C ± 2 °C with an equilibration time of 120 s in a DTS 1070 capillary cell. For these experiments, the attenuator position and intensity were set automatically. The samples were prepared using ~130 mg of the emulsified system, which was diluted in 20 mL of ultrapure water and manually stirred. From this, a 50 µL aliquot was taken and diluted with 1 mL of ultrapure water before each zeta potential measurement. Conversely, the electrical conductivity and the pH were determined using a conductivity meter and pH meter (Hanna Edge, Hanna Instruments, Woonsocket, Rhode Island, USA), respectively. All measurements were performed in triplicate.

### 2.5. Analytical Methodology for Drug Quantification

#### 2.5.1. Stock Solutions and Standards 

Stock solutions of CBZ with a final concentration of 1 mg/mL were prepared in triplicate, where 50.0 mg of the drug was dissolved in 50 mL of methanol. Similarly, the internal standard solutions (1 mg/mL) were prepared using propyl-paraben. Briefly, 50.0 mg of propyl-paraben was dissolved in 50 mL of methanol. Regarding the in vitro and in vivo release studies, calibration curves of CBZ with concentrations of 2.5, 5.0, 10.0, 20.0, and 100.0 µg/mL were developed, which included the internal standard with a fixed concentration of 10 µg/L. In the case of in vitro studies, methanol was used as solvent, whereas for in vivo studies, rabbit plasma was utilized as the dissolution matrix.

#### 2.5.2. Equipment and Chromatographic Conditions 

The quantification of CBZ was conducted via HPLC (Lachrom elite, Merck, USA) equipped with a photo diode array (PDA) detector and an automatic sampling system. The mobile phase consisted of methanol and water (40:60), and the flow rate was 1 mL/min. Separation was achieved using a 50 mm × 4.6 mm, Zorbax Eclipse XDB-C18 (Agilent technologies, USA) reversed-phase column with an average particle size of 1.8 µm, keeping the column at 25 °C. The column effluent was monitored at 265 nm, and the chromatographic data analysis was performed with EZChrome software (Agilent technologies, USA).

### 2.6. In Vitro Drug Release 

The in vitro release of CBZ from the coarse emulsions and nanoemulsions was assessed by dialysis method, using 198 mL of a phosphate buffer pH 7.4 and 150 mM as aqueous medium under sink conditions. For this, volumes of 2 mL of the coarse emulsions and nanoemulsions were placed in dialysis sacs (Hi-media, mol. wt. cutoff 14kD) in triplicate and dialyzed at 37 °C for 24 h. Subsequently, the samples were taken from the external medium at intervals of 0.25, 0.50, 0.75, 1, 2, 3, 4, 6, 12, and 24 h. 

### 2.7. In Vivo Drug Release

New Zealand breed rabbits weighing between 2.30 and 3.80 kg (average weight 3.26 kg) were used in this study. The animals were bred in-house at the animal facility of Universidad Icesi (Cali, Colombia), in a controlled environment maintained at 25 °C ± 1 °C in separate cages with a 12 h/12 h dark/light cycle and received standard food and water ad libitum. The animals were handled in accordance with Colombian guidelines for animal welfare and care (Law 84/1989 and resolution 8430/1993). The study was conducted with the approval of the Institutional Animal Ethical Committee of the Universidad Icesi. Specific care was taken to minimize animal suffering and minimize the number of animals used. Eight animals were divided randomly into two experimental groups: Group 1 (Coarse emulsion + CBZ, *n* = 4) and Group 2 (Nanoemulsion + CBZ, *n* = 4). CBZ formulations were administered orally (12.5 mg/kg body weight) with the help of a Ryles tube after about 10 to 12 h of fasting. Blood samples (0.5 mL) were withdrawn from the left marginal ear vein. One blood sample was taken before drug administration, and the rest of the samples were taken at the intervals of 0.25, 0.50, 1, 2, 3, 4, 6, 12, 18 and 24 h following drug administration. The samples were centrifuged at 4000 rpm for 10 min at 4 °C to separate the serum. The separated serum was stored at −20 °C for further analysis by HPLC.

### 2.8. Statistical Analysis

The data were tabulated and analyzed using Microsoft Excel and Graph Pad Prism, respectively. The homogeneity of variance in the data was analyzed using Bartlett’s test. Statistical comparisons were made using a one-way ANOVA. The Bonferroni post-hoc test was used to determine significant differences between the two independent groups. A confidence level of 95% was adopted. Data are expressed as mean ± standard deviation.

## 3. Results

### 3.1. SIO/Water Interface Physicochemical Characterization

**Figure 2:**
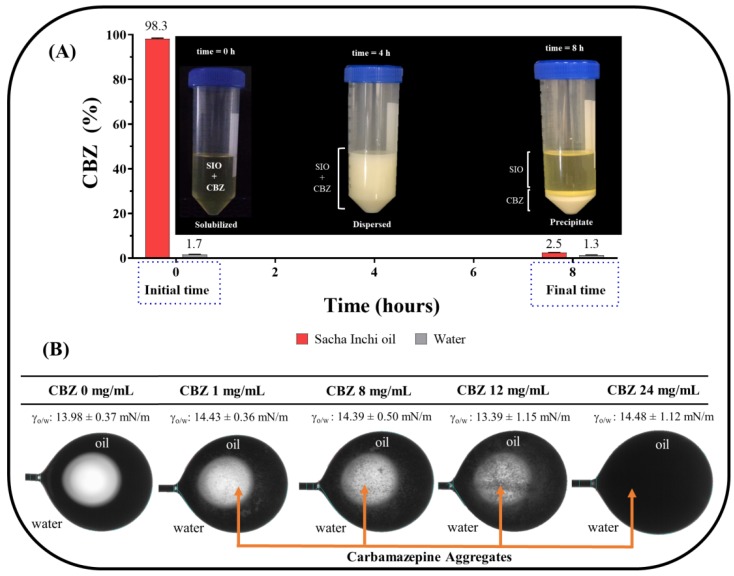
SIO/water interface physicochemical characterization. (**A**) SIO/water partition of CBZ with respect to time. (**B**) Effect of CBZ concentration inside oil droplets on interfacial tension.

The CBZ characterization studies between SIO, ultrapure water, and their respective interface are shown in Figure 2, where time-dependent different behaviors were observed. In the partition study performed, it was found that CBZ tended initially CBZ to be found mainly in the SIO (~98%), and small amounts were present in ultrapure water (~2%), which was consistent with the tricyclic nature of CBZ’s chemical structure that has a very low polarity [40]. After 8 h, CBZ migrated into the interface and precipitated, which left very little in either phase (oil or ultrapure water). This result was very interesting because it showed that CBZ thermodynamically favored the interface rather than the oily phase, as first observed at the initial time point (Figure 2A). 

Interestingly, the interfacial tension evaluation exhibited a very notable effect in that this parameter fluctuated slightly, with the inside of the oil droplet changing from a translucent appearance (typical of homogeneous systems) to a very opaque appearance (typical of heterodispersed systems) (Figure 2B). This result confirmed that CBZ was solubilized in the oily phase initially (with high agitation), but over time, the CBZ began to aggregate inside the oil droplet, changing from a solution to a suspension that migrated to the interfacial zone. These results are very significant for the design stage of emulsified formulations, since these indicate that CBZ will compete with the surfactants, and the physical stability could be affected as well as the drug release.

### 3.2. Elaboration of Emulsified Formulations

The coarse emulsions presented a slightly yellowish color with a sticky and partially rough texture, while the nanoemulsions showed an intense white color and a very soft or easy-to-spread texture. The initial physicochemical characterization data for freshly prepared coarse emulsions and nanoemulsions are summarized in Table 1.

### 3.3. Emulsion Stability Assay

**Figure 3:**
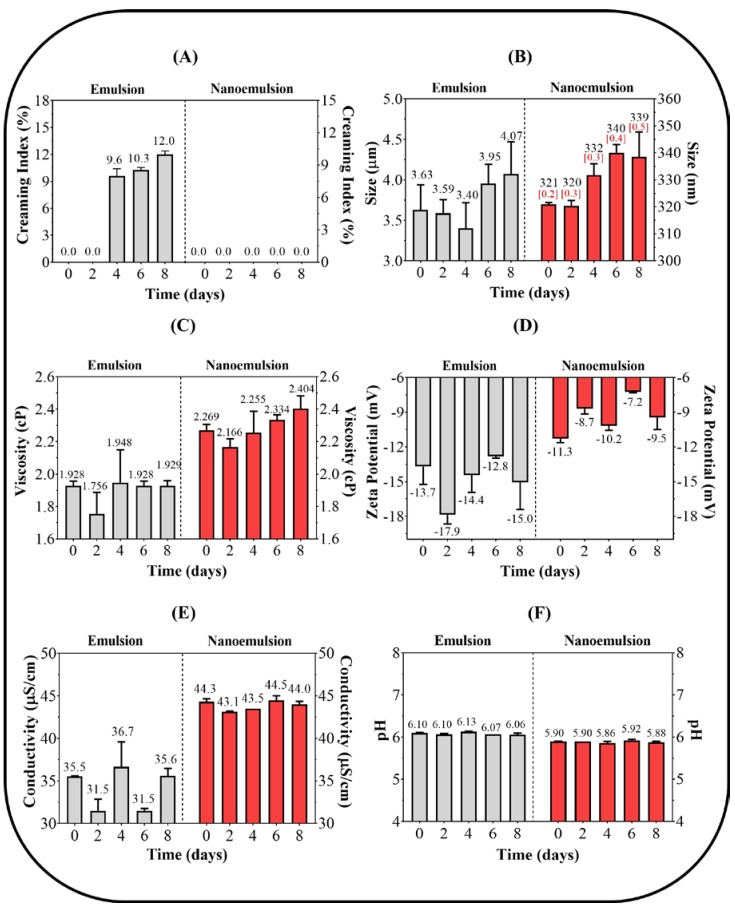
Results of (**A**) creaming index, (**B**) drop size, (**C**) viscosity, (**D**) zeta potential, (**E**) electrical conductivity, and (**F**) pH for coarse emulsion and nanoemulsion systems. In Figure 3B, the PDI values are presented in red brackets for the nanoemulsions only.

The results of the thermal stability for coarse emulsions and nanoemulsions are presented in Figure 3, where changes in the physicochemical parameters of creaming index (CI), droplet size, viscosity, zeta potential, conductivity, and pH with respect to time are described. Figure 3A shows that during the first 2 days of the study, there were no marked changes in the CI in either the coarse emulsions or the nanoemulsions. However, after 48 h, the coarse emulsions showed an increase in IC, reaching a maximum value of 12% after the second week, indicating a progressive decrease in the physical stability of the emulsion. In contrast, the nanoemulsions did not show any change in IC value, suggesting that they had greater physical stability. This result was consistent with the nanoemulsions being prepared by UHPH, where high shear energy was applied, leading to the formation of emulsified systems with very small droplet sizes and low PDI [36]. Thus, these characteristics demonstrate that nanoemulsions avoid the aggregation process between the dispersed droplets, thus providing a greater physical stability [37].

Conversely, as shown in Figure 3B, coarse emulsions and nanoemulsions tended to increase their droplet size over time. In the case of coarse emulsions, a change in droplet size from 3.63 ± 0.40 μm to 4.07 ± 0.60 μm was observed, while nanoemulsions changed from 320.9 ± 1.0 nm to 338.6 ± 8.0 nm. Additionally, the PDI of the nanoemulsions increased from 0.22 to 0.51. These data suggest that these systems changed from monodispersed to slightly polydispersed. These results took on more relevance when they are compared to the IC results, where smaller droplet size and PDI led to increased physical stability [25]. Contrarily, coarse emulsions with droplet sizes greater than 2 µm tended to have greater differential mobility between their droplets and tended to aggregate rapidly [41].

In relation to viscosity, Figure 3C shows that both emulsified systems had very fluid external phases, where coarse emulsions had values around 1.9 cP and nanoemulsions had values between 2.2 and 2.4 cP. This difference in the dispersed phase may be explained by the greater packaging degree and lower mobility of nanoemulsions, where the dispersed droplets were in a steady state, while the coarse emulsions were in a dynamic state [42].

Figure 3D shows that both emulsified systems had low zeta potential values that were indicative of a normal result when neutral surfactants were used (Tween ™ 80 and Span ™ 80). Likewise, it was noted that zeta potential values fluctuate between −12.82 ± 0.50 mV and −17.88 ± 1.10 mV and −7.23 ± 0.02 mV and −11.29 ± 0.50 mV for coarse emulsions and nanoemulsions, respectively. This difference could also be explained by the packaging degree and the stationary condition of the dispersed droplets in the nanoemulsions that leaned toward lower electrophoretic mobility, and therefore a lower zeta potential value [43].

In relation to the electrical conductivity in the external phase, Figure 3E shows the different behaviors of the emulsified system. For coarse emulsions, low and fluctuating values (31.5 and 35.5 µS/cm) were obtained, while for nanoemulsions, slightly higher and constant values were obtained (43.1 and 44.5 µS/cm). This behavior also supported the previous results, where nanoemulsified systems tended to form more organized monodispersed structures where the ionic species (preservatives) could move easily in the external phase of the emulsion. Finally, Figure 3F shows that coarse emulsions and nanoemulsions had pH values between 5.9 and 6.1. This result was consistent with acidic paraben preservatives [44]. Additionally, the pH values were almost constant, which suggested chemical integrity of the formulation ingredients.

### 3.4. Analytical Methodology for Drug Quantification

In accordance with the ICH standards [45], the results of the non-exhaustive validation for the analytical methods of CBZ quantification are summarized in Table 2.

The results in Table 2 show that the correlation coefficients values for the calibration curves in in vitro and in vivo studies were 0.9973 and 0.9997, respectively. The calibration curves complied with the linearity validation parameter and the data obtained fitted very well to the heuristic model, *y = mx + b*, where *y* is the analytical signal, *m* is the line slope, *x* is the CBZ concentration in units of µg/mL and *b* is the intercept. Likewise, it was found that CBZ detection and quantification limits presented a 10-fold concentration difference factor, and the analytical method reproducibility, expressed as the RSD, was always less than 5%.

### 3.5. In Vitro Drug Release

**Figure 4:**
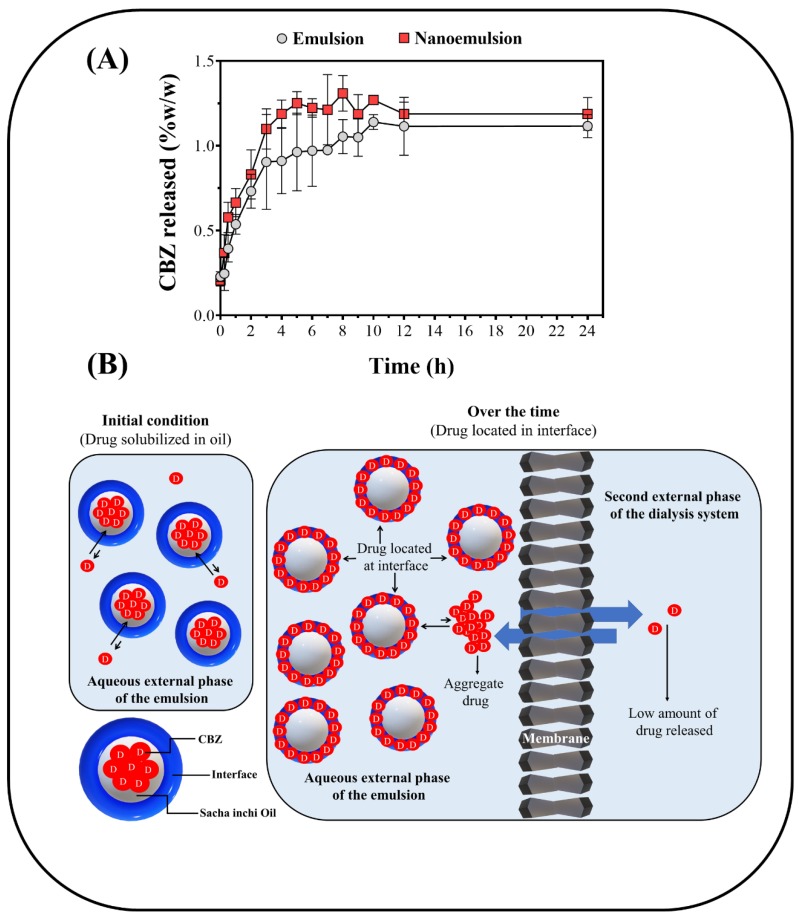
(**A**) In vitro CBZ release for emulsion and nanoemulsion formulations. (**B**) Scheme of CBZ migration from the oily phase to the interfacial zone, affecting the drug’s permeability.

Figure 4A shows the in vitro release profiles of CBZ from coarse emulsions and nanoemulsions. Both emulsions had a rapid release (burst release) in the first hours, followed by a deceleration [46]. It is important to note that CBZ release was extremely low (<2%) in the coarse emulsions as well as nanoemulsions at very early time points. The CBZ release was lower than anticipated based on the results of previous publications examining emulsified CBZ systems [47,48,49,50,51]. However, the previous studies examined CBZ release under different conditions: (i) CBZ in lower amounts, (ii) different types of oil phase, (iii) higher concentrations of surfactants, and (iv) use of dialysis membranes with large cutoff sizes. In contrast, in this study, a high amount of CBZ was used with a minimum quantity of surfactants (2%) to guarantee that the drug was vehiculized in the emulsified systems and not in other types of colloidal structures like micelles. In addition, dialysis membranes with smaller cutoff size were employed (14 kDa) to more closely mimic actual biological conditions [52,53]. To further expound upon the low amount of CBZ released from both emulsification systems, it is necessary to consider the drug characterizations made in previous studies with SIO, ultrapure water, and the respective interface. First, the SIO/water partition and the interfacial tension evaluations showed that CBZ has a very poor solubility in both emulsion phases (oily and aqueous), and spontaneously tends to both precipitate and migrate to the interface. Thus, the CBZ diffusion into the aqueous phase of the emulsion (external phase) is considerably limited. It is limited to an even greater degree in the second aqueous external phase of the two-compartment dialysis system (Figure 4B). Second, taking into account the larger pore size of the dialysis membrane used in the previous studies, and the nature of CBZ forming aggregates and heterogeneous dispersions in aqueous medium, it is possible to understand how our results, which employed dialysis membranes with smaller pore sizes, led to a lower CBZ release from both emulsified systems. However, it is important to highlight that these results of in vitro carbamazepine release, instead of providing clarity about its release mechanism from such emulsified systems, generated more doubts, which should be considered in subsequent investigations. Thus, the biopharmaceutical classification system of the drug, the load of drug in the emulsions, the membrane type, the membrane cut-off, and the pH of the medium, especially at levels that simulate the gastrointestinal tract (1.2; 4.5 and 6.8), should be considered and studied in more depth to clarify these in vitro release mechanisms.

### 3.6. In Vivo Drug Release

**Figure 5:**
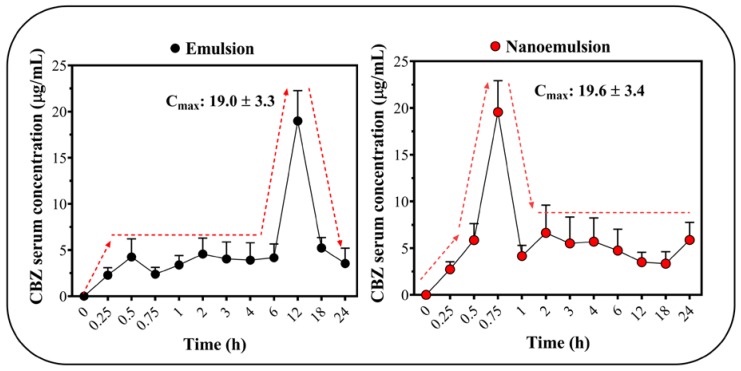
CBZ blood concentration released from coarse emulsion and nanoemulsion formulations.

The results of CBZ in vivo release are presented in Figure 5. A 10-fold increase in drug concentration was found in vivo compared to the in vitro release studies. The maximum plasma concentration (C_max_) of CBZ was ~19 µg/mL in the coarse emulsions and nanoemulsions (Figure 5), which corresponded to 20% *w*/*w* of CBZ absorbed. Although these results were similar to the plasma CBZ concentrationd previously found in other animal models and in other dosage forms [54], it is necessary to highlight that these results differed greatly from the usual reported values for this drug (>60% *w*/*w*) [55]. It was also observed that the differences between the droplet sizes of the emulsified formulations did not significantly affect CBZ plasma concentration; however, it should be noted that the time in which the maximum plasma concentrations (t_max_) was reached depended considerably on the type of emulsified system. In the case of coarse emulsions, it was found that the t_max_ was 12 h. The t_max_ for nanoemulsions was found to be 45 min, and this corresponded to a considerable change in the drug absorption rate, which is a remarkable and promising result. These results suggest that nanoemulsified formulations may have different absorption mechanisms, where smaller droplets can more easily permeate the gastrointestinal wall and endothelial membranes, thereby rapidly achieving the desired concentration in the blood plasma, and this might have the potential to increase bioavailability.

These results confirm that nanoformulations of conventional drugs are very promising alternatives to traditional drug delivery methods and continue to further the research into improving the physicochemical and biopharmaceutical features of conventional pharmaceuticals.

## 4. Conclusions

CBZ showed a low affinity for the oily and aqueous phases and tended to spontaneously migrate toward the interfacial zone over time. UHPH resulted in a remarkable improvement in the sensorial characteristics of the nanoemulsions, as well as the physical stability of the coarse emulsions, leading to the creation of emulsified formulations with an intense white appearance and a soft texture due to their small and monodispersed droplets (~320 nm and PDI < 0.3). The release of CBZ in vitro was low for both emulsified formulations (<2% *w*/*w*), which was attributed to the remarkable predilection of the drug for the SIO/water interface, affecting its diffusion to the emulsion external phase as well as its diffusion to the second aqueous phase in a two-compartment dialysis system. However, these results are not conclusive, and it is therefore necessary to carry out more studies focused on clarifying this behavior. Thus, the effect of the drug load on the emulsion, the type of membrane, the pore size of the membrane, and the pH of the gastrointestinal medium with respect to the drug release should be studied. In contrast, the in vivo CBZ release study had a maximum plasma concentration 10-fold higher than the in vitro study, which corresponded to ~20% *w*/*w*. Although the concentration values typically reported for orally administered CBZ (>60% *w*/*w*) are greater than those obtained in this study, the maximum absorption times were considerably decreased in this study. The time to absorption of CBZ is dependent on the formulation. CBZ nanoemulsions were able to dramatically decrease the time to maximum absorption, from 12 h with the coarse emulsions to just 45 min, indicating a great improvement in bioavailability. These data show that nanoformulations provide a rich and fertile research landscape for the development of innovative dosage forms of conventional pharmaceutical drugs.

## Figures and Tables

**Table 1:** Physicochemical characterization of freshly prepared oil-in-water coarse and nanometric emulsions.

Physicochemical Parameter	Coarse Emulsion	Nanoemulsion
Droplet size	3.63 ± 0.40 µm	320.90 ± 1.00 nm
Viscosity	1.93 ± 0.02 cP	2.27 ± 0.05 cP
Zeta potential	−13.69 ± 1.20 mV	−11.29 ± 0.20 mV
Conductivity	35.50 ± 0.50 µS/cm	44.30 ± 0.70 µS/cm
pH	6.10 ± 0.05	5.90 ± 0.06

**Table 2:** Validation of non-exhaustive analytical method for CBZ quantitation.

Parameter	In Vitro Correlation Curve	In Vivo Correlation Curve
Linear equation X (µg/mL)	Y = 0.026X + 0.0875	Y = 0.042X + 0.0219
Correlation coefficient	0.9973	0.9997
Detection limit (µg/mL)	0.015	0.020
Quantitation limit (µg/mL)	0.15	0.20
Intra-day method RSD (%)	0.40	1.40
Inter-day method RSD (%)	1.59	2.40

RSD: residual standard deviation.
